# Anxiety and Attentional Processes: The Role of Resting Heart Rate Variability

**DOI:** 10.3390/brainsci11040480

**Published:** 2021-04-09

**Authors:** Giuseppe Forte, Francesca Favieri, Esther Osariemen Oliha, Andrea Marotta, Maria Casagrande

**Affiliations:** 1 Dipartimento di Psicologia, Università di Roma “Sapienza”, 00185 Rome, Italy; francesca.favieri@uniroma1.it (F.F.); estheraphex@gmail.com (E.O.O.); 2 Departamento de Psicología Experimental, Universdad de Granada, 18012 Granada, Spain; marotta@ugr.es; 3 Dipartimento di Psicologia Dinamica, Clinica e Salute, Università di Roma “Sapienza”, 00185 Rome, Italy

**Keywords:** anxiety, attention, emotion, flicker task, change blindness, heart rate variability (HRV)

## Abstract

Individuals with high anxiety preferentially focus attention on emotional information. The autonomic nervous system (ANS) plays an important role in modulating both anxiety and attentional processes. Despite many studies having evaluated attentional bias in anxious people, few of them have investigated the change blindness phenomenon associated with the attentional response toward salient stimuli, considering the role of the ANS. This study aimed to examine the role of heart rate variability (HRV) in trait anxiety and top-down and bottom-up attentional processes toward emotional stimuli. Seventy-five healthy university students were divided into high (*N* = 39) and low (*N* = 36) trait anxiety groups and completed a change detection flicker task with neutral, positive, and negative stimuli. The results evidenced a different attentional pattern between people with high and low anxiety considering both the two attentional processes and the valence of the stimuli. Specifically, individuals with high anxiety showed a bias in elaborating emotional stimuli related to their salience (i.e., negative stimuli were faster elaborated than neutral and positive stimuli when top-down attentional mechanisms were involved, while slower performances were highlighted considering bottom-up attentional mechanisms in response to emotional stimuli compared to neutral stimuli). Moreover, an association between HRV, trait anxiety levels, and change blindness phenomenon was confirmed. These results underline the role of HRV as a possible predictor of the alteration of attentional mechanism in anxiety.

## 1. Introduction

Anxiety is an organic and psychological response, characterized by apprehension and increased surveillance in situations of uncertain danger or potential threats to the organism’s integrity [1,2]. Individuals with high anxiety preferentially focus on negative information [3,4], with possible alterations in attentional control due to threatening stimuli [5]. Attentional deficits seem to constitute one of the most relevant cognitive vulnerability factors for anxiety [6], highlighting a bidirectional interaction between anxiety and attention. Many cognitive models implicate attentional dysfunctions toward threatening stimuli in the genesis and maintenance of anxiety [7]. The hypervigilance toward threatening stimuli than neutral information [4] can be considered as a component of the attentional bias (AB), indicating a dysfunction in the information processing [8,9]. AB is often described as an adaptive neurocognitive function useful to identify and respond to an upcoming threat [10]. AB occurs when attention is preferentially directed toward an emotionally salient stimulus [11], and many studies have focused on the role it can play in the etiology and maintenance of anxiety [3,4]. Previous results on AB in anxiety are inconsistent [12]; some studies evidenced in high anxiety condition an AB toward threat stimuli, while other authors reported an AB away from threat stimuli [13,14,15]. These inconsistencies could be due to both the difficulty in conceptualizing the attentional process of emotional information and the heterogeneity in the experimental designs used to evaluate AB. In fact, investigations on AB in anxiety were influenced by (1) the paradigm adopted for its assessment (e.g., dot-probe paradigm); (2) the characteristics and nature of the stimuli (e.g., picture, words); (3) the samples’ characteristics (i.e., pathological samples, subclinical samples); and (4) the manipulation of variables (i.e., anxiety induction) [12]. To overcome these limitations and clarify the relationship between anxiety and AB, further studies are needed.

An important role in both anxiety and attentional processes is played by the autonomic nervous system (ANS). A non-invasive method to evaluate the ANS activity is the measure of heart rate variability (HRV). HRV is defined as an index of autonomic heart control and reflects the time interval variations between consecutive heartbeats resulting from parasympathetic vagal inputs through the sinoatrial node [16,17].

Polyvagal theory [18] and neurovisceral integration model [19] have proposed that HRV is associated with the activity of the prefrontal cortex that, through the vagus nerve, connects the heart and brain, suggesting that individual differences in HRV may be due to differences in prefrontal cortex activity [19]. This model indicates that the prefrontal-subcortical inhibitory circuit is fundamental for self-regulation [20,21], supporting the individual’s adaptive and flexible response to environmental demands [19,20]. In this context, low HRV levels are associated with worse cognitive regulation, resulting in cognitive biases and, in general, in a more rigid cognitive pattern [19,20]. In contrast, high levels of HRV are associated with more effective emotional and attentional regulation strategies and, in general, determine adequate cognitive performance including an attentional one (for a review, see [22]). Moreover, a recent systematic review [23] suggested a reduced ANS activity in attentional dysfunction, characterized by an ANS hypoactivation during resting state. Although some studies have underlined the association between anxiety and HRV [24,25], nobody has evaluated it in relation to AB related to anxiety.

### Aims

This study aimed to investigate the association between trait anxiety levels and attentional mechanisms. Attentional bias was analyzed by adopting the change detection flicker task, which is based on the change blindness phenomenon [26,27]. The flicker task measures attentional bias for salient target stimuli that capture attention including stimuli that can trigger anxiety. It is based on alternating visual scenes of real life, differing only for modified detail (A→A′). This alternation is carried out until the identification of the change. The experimental paradigm principle is that individuals usually detect changes in the central interest (CI) area of a scene faster than changes in the marginal interest (MI) area. Due to the movement of focused attention in the environment, the flicker procedure helps analyze both automatic (bottom-up) and voluntary (top-down) components of attention [28,29]. In the ecological context, the salience of a visual stimulus influences the automatic orienting of attention, and subject motivation drives the voluntary orienting of attention [30]. Recently, this paradigm has been used to measure attentional bias in many conditions including anxiety disorders [31]. In this study, we adopted emotional (both positive and negative) and neutral pictures representative of a real context to evaluate the role of the valence of the stimuli in the attentional bias in individuals with different anxiety levels.

Accordingly, it was assumed that:

- Given the higher arousal associated with anxiety [32], greater trait anxiety should be related to larger attentional bias for the change detection in a flicker paradigm, regardless of the emotional value of the stimulus. The ability to detect changes was expected to be greater for negative stimuli. In fact, according to previous studies, individuals with high trait anxiety would seem to focus on negative information [4] preferentially and would present greater difficulties, compared to subjects with low trait anxiety, to disengage themselves from threatening information [33].
- Individuals with high trait anxiety should present a reduced vagal tone, in line with previous results in clinical and subclinical populations [24,25,34].
- HRV should be associated in attentional processes related to the change blindness paradigm and anxiety.

## 2. Materials and Methods

### 2.1. Participants

From an initial sample of 173 university students recruited voluntarily, participants were selected according to their trait anxiety levels. High trait anxiety scores (above the 80th percentile) and low trait anxiety scores (below the 20 percentile) [35] were obtained by measuring trait anxiety with a standardized questionnaire (State-Trait Anxiety Inventory) [36]. Participants’ exclusion criteria were a diagnosis of psychopathologies, head trauma, use of drugs, sleep disorders, or chronic medical conditions (e.g., cardiovascular pathologies, metabolic syndromes, hormonal dysfunctions). All participants had normal or correct-to-normal visual acuity and absence of color blindness. The final sample included 75 participants (aged between 18–30 years) divided into: high trait anxiety (HTA; *N* = 39, mean STAI scores = 55.5; SD = 5.0) and low trait anxiety (LTA; *N* = 36, mean STAI scores = 28.2; SD = 2.9). The participant flow-chart regarding the exclusion process is reported in the Supplementary Materials.

### 2.2. Measurements

#### 2.2.1. Self-Report Measures

The trait scale of the State-Trait Anxiety Inventory (STAI) [36,37] is a self-assessment questionnaire to evaluate state and trait anxiety on a 4-point Likert scale (1 = not at all; 4 = very much). Higher scores on the STAI indicate greater anxiety levels. Considering the aim of the study, only trait anxiety (20 items) was assessed.

#### 2.2.2. Emotional Flicker Task

The emotional flicker task was adopted to evaluate the change blindness [29].

##### Apparatus

The task was administered through a Personal Computer with a 19″ high definition monitor. The administration of stimuli and response time recordings were programmed using E-Prime 2.0 software on an Intel Core i5 PC. Responses were collected via a computer keyboard.

##### Stimuli

**Figure 1:**
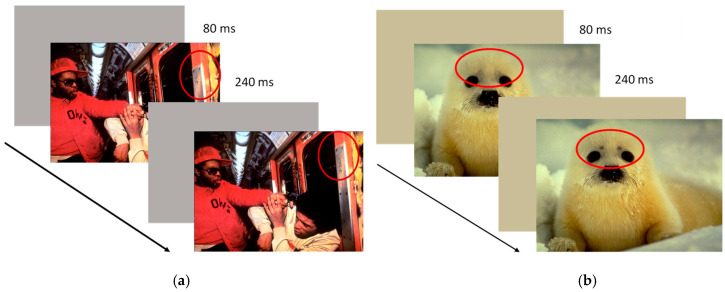
Examples of flicker task trials. (**a**) Marginal interest change in negative scene. (**b**) Central interest change in positive scene.

Twenty-four pictures from the IAPS (International Affective Picture System; [38]), were selected on the basis of their emotional valence: eight pictures (9253, 9433, 3500, 2205, 9410, 3530, 9254, 6520) with negative valence (mean valence score = 1.92 ± 0.20; mean arousal score = 6.18 ± 0.87), eight pictures (7550, 2102, 7036, 2026, 7130, 5471, 2411, 2308) with neutral valence (mean valence score = 4.99 ± 0.25; mean arousal score = 3.47 ± 0.31), and eight pictures (5833, 2080, 1710, 1460, 2340, 1440, 1750, 2154) with positive valence (mean valence score = 8.17 ± 0.11; mean arousal score = 4.32 ± 1.35). The pictures (640 × 480 pixels) were manipulated via Photoshop software (ver. CS6-13.0) to have two alternative versions of them identical except for a detail removed by one of the scenes (49 × 49 pixels). According to the procedure indicated by Rensink [26], half of the pictures for each valence showed a change of central interest (CI), and half of the pictures showed a change of marginal interest (MI) (see Figure 1). The central and marginal interest changes were generated considering the subjective evaluations of the pictures by an independent group of 17 undergraduate students (average age 22.38; SD = 3.45). Participants were required to observe the pictures for 3 s and to report and spatially indicate on a grid for each picture which item was first identified. The items mentioned by more than 90% of the subjects were considered of central interest; the items chosen by no more than two participants were defined as marginal interest.

##### Procedure

Participants were individually tested in a quiet, dimly lit room. Each participant was seated at approximately 56 cm from the PC screen. The two pictures (the original and the modified ones) were reproduced in rapid sequence alternated by a blank screen. Figure 1 shows an example of the procedure. The item duration was 240 ms, and the blank screen duration was 80 ms [39]. When the participants identified the change, they had to press the space bar of the keyboard and indicate the change. At the beginning of the experiment, three trials were proposed (practice trials). Subsequently, they had to complete three randomized experimental blocks according to the stimuli valence (negative, positive, neutral).

#### 2.2.3. Physiological Measures

The following physiological measures were recorded:

- Blood pressure: Blood pressure was measured using an OMRON digital sphygmomanometer in line with the European guidelines for measuring blood pressure [40]. Systolic and diastolic blood pressure and heart rate were collected.
- Weight and height: A digital balance was used to measure the participants’ weight (kg). The height of the subjects was measured with a standardized anthropometer (cm). These data allowed the assessment of the body mass index (BMI: kg/m^2^).
- Heart rate variability: Electrocardiography (ECG) was recorded using disposable Ag/AgCI electrodes through the Firstbeat bodyguard 2. Two electrodes were positioned on the right side of the body, above the clavicle, and on the left side, at the rib cage level. HRV was analyzed using the Kubios HRV program, and artifacts were manually and automatically removed (ver. 2) [41]. The results in the time domain and the frequency domain were analyzed. In the time domain, in line with previous studies and according to guidelines, given the high correlation among HRV indices, only the root mean square of successive differences (RMSSD) that reflects vagal tone [20,42,43] was considered. In the frequency domain, the low-frequency range (LF; 0.04–0.15 Hz) that reflects a mix of sympathetic and vagal influences [16,42] and high frequencies (HF; 0.15–0.40 Hz), an index of the parasympathetic cardiac tone [17,42,44], were considered.

#### 2.2.4. General Procedure

**Figure 2:**
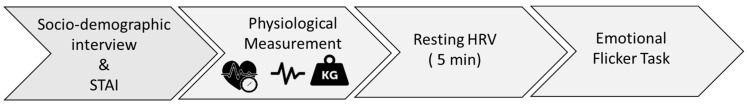
General procedure. STAI: State-Trait Anxiety Inventory; HRV: Heart Rate Variability.

The protocol was approved by the Ethics Committee of the Department of Dynamic and Clinical Psychology and Health Studies of the University of Rome “La Sapienza” (Protocol number: 0001166-21/08/2019), and all participants signed the informed consent. According to the international guidelines for the assessment of HRV [44], participants were required to avoid tobacco, smoke, and caffeine or their consumption in the two hours preceding the evaluation as well as alcohol consumption and intensive physical activity in the 12 h preceding the experiment. After completing the State-Trait Anxiety Inventory (STAI), weight and height were measured, and blood pressure was recorded. Then, HRV in the resting phase was recorded for five minutes while the participants sat with knees at a 90° angle, both feet flat on the floor, hands on thighs, with palms facing upward, and eyes closed [44]. Finally, they completed the emotional flicker task (Figure 2).

#### 2.2.5. Data Analysis

To evaluate group differences in the demographic, clinical, and cognitive variables, one-way analysis of variance (ANOVA), considering the group (HTA; LTA) as a between variable, was computed on each of the following variables: age, BMI, systolic and diastolic blood pressure, heart rate, STAI score, and HRV indices. A chi-square comparison was used to test differences in sex distribution. If significant differences between groups in BMI or age were found, these measures were considered confounding variables and controlled in statistical analysis.

For the emotional flicker task, according to Maccari et al. [29], the response times (RTs) in incorrect responses were replaced by the mean RTs + 2.5 SD for each condition (considering change type and stimulus valence). All participants showed a percentage of accuracy greater than 50%. Performance in the flicker task was evaluated by a 2 × 2 × 2 mixed ANOVA design with the group (HTA; LTA) as a between-subject variable and type of change (CI, MI), and stimulus valence (emotional; neutral) as within-subject variables; RTs in the flicker task were considered as the dependent variable. Moreover, to further deepen the role of emotional valence and to differentiate the role of positive and negative stimuli, a 2 × 2 × 3 mixed ANOVA design considering the group (HTA; LTA) as a between-subject variable, the type of change (CI, MI), and stimulus valence (positive; negative; neutral) as within-subject variables, and RTs as the dependent variable, was conducted. Planned comparisons were considered to investigate the significative effects and interactions further.

Considering HRV, RMSSD (ms) for the time domain and HF (ms2) and LF (ms2) for the frequency domain were adopted for the statistical analyses. Since HRV measures in absolute units (ms2) are usually positively skewed, HRV indices were subjected to a natural log (ln) transformation to normalize the distribution. Pearson’s r correlations were calculated to detect the association between HRV indices and RTs in the emotional flicker task. In addition to the RTs for each condition of the task, two indices for CI changes (mean RTs of negative, positive, and neutral scenes in which the changes were of CI) and MI changes (mean RTs of negative, positive, and neutral scenes in which the changes were of MI) were calculated. In order to highlight associations with the HRV index between variables, Pearson correlations were conducted separately for each group. Differences between mean scores were expressed as effect sizes with pη^2^ (partial variance explained for each independent variable). Associations were expressed in Pearson’s r. Variables were normally distributed (Shapiro–Wilk test *p* > 0.05), and Levene’s tests confirmed homoscedasticity in all cases (*p* > 0.05). Statistical analyses were performed using SPSS-25, the level of significance was accepted at *p* < 0.05.

## 3. Results

### 3.1. Demographic variables

The ANOVAs did not show significant differences between the groups for age, BMI, systolic and diastolic blood pressure, and heart rate (all F < 3.15; all *p* > 0.08). Moreover, no differences were found in HRV indices in the resting state between groups (all F < 1.96; all *p* > 0.16). A percentage of 9.3% of participants (seven out of 75), reported STAI score above two standard deviation from the average score of normative data. Table 1 shows the principal characteristics of the participants.

#### 3.1.1. Flicker Task: Attentional Effects

A significant main effect of the Type of Change (F_1,74_ = 477.31; *p* = 0.0001; pη^2^ = 0.86) was evidenced. Overall participants detected faster CI than MI changes (mean difference: −17,515 ms). The main effects of the Stimulus Valence (F_1,74_ < 1; *p* = 0.65) was not significant. The Type of Change × Stimulus Valence interaction (F_1,74_ = 10.63; p = 0.002; pη^2^ = 0.13) was significant, highlighting faster detection of CI changes than MI changes for both emotional (mean difference: −15,091 ms; F_1,74_ = 271.66; *p* = 0.0001) and neutral (mean difference: −19,939 ms; F_1,74_ = 256.15; *p* = 0.0001) pictures. Moreover, while faster detection of CI changes was shown in neutral stimuli than emotional stimuli (mean difference: −2072; F_1,74_ = 23.31; *p* = 0.0001), the opposite pattern was reported for the detection of MI changes (mean difference: 2775; F_1,74_ = 3.57; *p* = 0.05).

These results were confirmed considering individually positive and negative valence of the stimuli. The significant main effect of Stimulus Valence (F_2,144_ = 9.45; *p* = 0.0001; pη^2^ = 0.12) revealed a faster detection time in negative stimuli compared to both neutral (mean difference: −4417; F_1,74_ = 6.78; *p* = 0.02) and positive (mean difference: −7647; F_1,74_ = 20.39; *p* = 0.0001) stimuli. No differences were highlighted between neutral and positive stimuli (mean difference: −3231; F_1,74_ = 2.89; *p* = 0.10). Moreover, in the *Type of Change* x *Stimulus Valence* (F_2,144_ = 23.83; *p* = 0.0001; pη^2^ = 0.25) the detection of CI changes was faster in neutral stimuli than both negative (mean difference: −2796; F_1,73_ = 20.75; *p* = 0.0001) and positive (mean difference: −1432; F_1,73_ = 12.51; *p* = 0.0001); in contrast, the detection of MI changes was faster in negative than both neutral (mean difference: −7212; F_1,73_ = 25.09; *p* = 0.0001) and positive (mean difference: −9012; F_1,73_ = 32.51; *p* = 0.0001) stimuli.

#### 3.1.2. Flicker Task: Group Differences (Neutral vs. Emotional Stimuli)

The main effects of the Group (F_1,74_ < 1; *p* = 0.90) and the interaction *Group × Type of Change* (F_1,74_ < 1; *p* = 0.90) were not significant.

**Figure 3:**
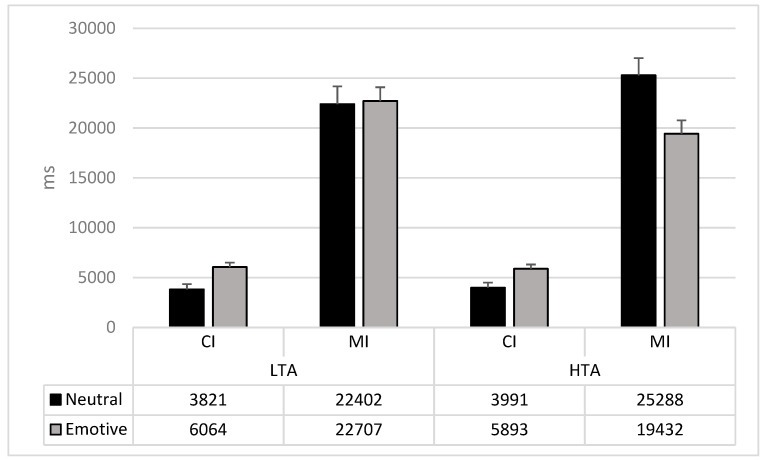
Mean and Std.Err of the interaction Group × Type of Change × Emotion. CI: Central interest changes; MI: Marginal interest changes; LTA: Low trait anxiety group; HTA: High trait anxiety group.

The Group × Stimulus *Valence* (F_1,74_ = 4.29; *p* = 0.04; pη^2^ = 0.06) and the Group × Type *of* Change × Stimulus Valence (F_1,74_ = 3.84; *p* = 0.04; pη^2^ = 0.05) interactions were significant. Specifically, the HTA group showed a faster detection time of CI changes in neutral than emotional stimuli (mean difference: −1901; F_1,74_ = 10.09; *p* = 0.002) and faster detection of MI changes in emotional stimuli than neutral stimuli (mean difference: −5856; F_1,74_ = 8.18; *p* = 0.005; see Figure 3). In LTA, only the detection of CI changes significantly differed between neutral and emotional stimuli (mean difference: −2245; F_1,74_ = 13.30; *p* = 0.0005) with faster detection of neutral stimuli than the emotional one. No differences emerged for MI changes (F_1,74_ < 1; *p* = 0.89; see Figure 3 and Table 2).

#### 3.1.3. Flicker Task: Group Differences (Neutral vs. Positive vs. Negative Stimuli)

Considering individually positive and negative stimuli, the Group × Stimulus Valence interaction (F_2,144_ = 3.74; *p* = 0.02; pη^2^ = 0.05) revealed between-group differences underlining that the HTA group were faster at detecting changes in positive scenes (mean difference: −6469; F_1,74_ = 3.99; *p* = 0.05) compared to the LTA group. Within-differences in the HTA group indicated faster detection times of negative than both positive (mean difference: −4777; F_1,73_ = 4.15; *p* = 0.05) and neutral (mean difference: −6342; F_1,73_ = 7.29; *p* = 0.01) stimuli. In the LTA group, a faster detection time of negative stimuli than positive (mean difference −10,518 ms; F_1,73_ = 18.52; *p* = 0.0001) was highlighted, but no difference in the detection time of negative and neutral stimuli (mean difference: −8028; F_1,73_ = 8.56; *p* = 0.005).

The Group × Type of Change × Stimulus Valence (F_2,144_ = 3.93; *p* = 0.02; 0.05) interaction showed that the HTA group detected MI changes of positive scenes faster than the LTA group (mean difference: −6467; F_1,73_ = 4.49; *p* = 0.04).

Both groups showed the same pattern in the detection of CI changes, with faster response time for neutral than negative (LTA: mean difference: −3132; F_1,73_ = 12.51; *p* = 0.001; HTA: mean difference: −2458; F_1,73_ = 8.37; *p* = 0.005) and positive stimuli (LTA: mean difference: −1518; F_1,73_ = 6.76; *p* = 0.01; HTA: mean difference: −1344; F_1,73_ = 5.75; *p* = 0.02), and no differences considering positive and negative stimuli (LTA: mean difference: 1614; F_1,73_ = 3.80; *p* = 0.07; HTA: mean difference: 1115; F_1,73_ = 1.97; *p* = 0.16).

**Figure 4:**
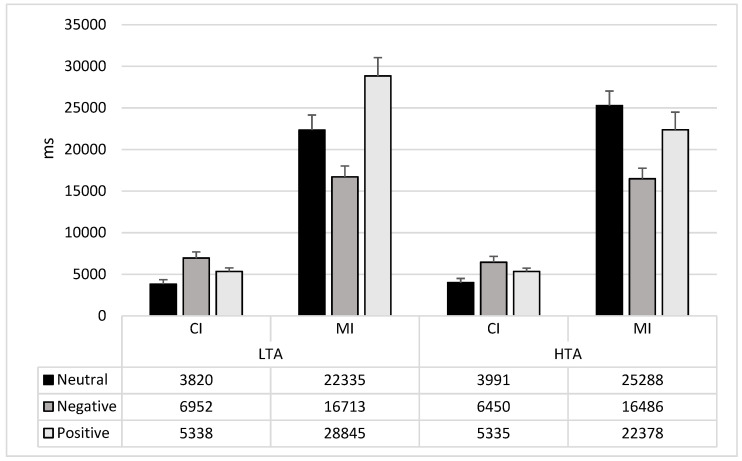
Mean and Std.Err of the interaction Group × Type of Change × Emotion. CI: Central interest changes; MI: Marginal interest changes; LTA: Low trait anxiety group; HTA: High trait anxiety group.

Considering the detection of MI changes, some differences emerged. Both LTA and HTA groups showed faster response time for negative stimuli than for both neutral (LTA: mean difference: −5623; F_1,73_ = 7.32; *p* = 0.01; HTA: mean difference: −8802; F_1,73_ = 19.48; *p* = 0.0001) and positive stimuli (LTA: mean difference: −12132; F_1,73_ = 28.30; p = 0.0001; HTA: mean difference: −5892; F_1,73_ = 7.24; *p* = 0.01). However, the LTA group reported faster detection of the MI changes for the neutral than positive stimuli (mean difference: 6509; F_1,73_ =5.65; *p* = 0.02), while no difference was highlighted in the HTA group (mean difference: 2910; F_1,73_ = 1.23; *p* = 0.27) (see Figure 4 and Table 3). 

### 3.2. Anxiety, Emotional Flicker Task, and Heart Rate Variability (HRV)

In the overall sample (i.e., without taking into account anxiety), significant negative association was highlighted between both LF (ln) and HF (ln) and the detection time of CI changes (LF (ln): *r* = −0.24; *p* = 0.04; HF (ln): *r* = −0.28; *p* = 0.02). Moreover, HF (ln) was negatively associated with the detection time of CI changes in neutral scenes (*r* = −0.26; *p* = 0.03).

When these correlations were examined as a function of the anxiety, different patterns were highlighted. In the HTA group, negative correlations emerged between RMSSD (ln), LF (ln), and HF (ln) and the detection time of CI changes (RMSSD (ln): *r* = −0.36; *p* = 0.03; LF (ln): *r* = −0.30; *p* = 0.08; HF (ln): *r* = −0.40; *p* = 0.02), and these associations were confirmed by considering RMSSD (ln) and (HF (ln) and the detection time of CI changes in neutral scenes (RMSSD (ln): *r* = −0.32; *p* = 0.06; HF (ln): *r* = −0.35; *p* = 0.04). In contrast, in the LTA group, no significant correlation between HRV indices and RTs in the emotional flicker task was found (see Table 4).

## 4. Discussion

The current study aimed to investigate the association between anxiety, attentional processes, and HRV. In particular, the study was designed to measure the moderating role of HRV in the change blindness phenomenon for emotional stimuli in healthy young individuals with subclinical high trait anxiety. Several studies showed that HF-HRV moderates attentional processing of threatening or emotional-salient stimuli [45,46] and modulates the influence of trait anxiety on attentional processing for emotion-driven stimuli [35,47,48]. Our results evidenced an association between HRV, trait anxiety levels, and change blindness phenomenon, as assessed by the emotional flicker task.

According to previous studies, the analyses of the performance in the emotional flicker task confirmed the main effect of the paradigm. Faster detection of changes in central interest areas than marginal interest areas of the scene [26] was evidenced. Central interest changes generated a pop-out effect, leading to an automatic capture of attention and a bottom-up attentional elaboration to detect changes. Marginal interest changes were detected via a serial visual search strategy (i.e., exploring the overall scene) adopting top-down processing [28,29,49], which requires a longer time than bottom-up processing. Considering the emotional valences of scenes, faster detection of changes in negative scenes emerged, suggesting a presence of a negative bias. However, considering the change type, this bias was consistent only in detecting marginal interest changes. These results can be subject to different interpretations. A first explanation can be ascribed to the nature of the bottom-up attentional process toward emotional stimuli. The emotional valence of a scene would cause a difficulty to disengage the attention from the attentional focus in the early and more automatic phases of the attentional process [50], delaying the detection time of a change in the scene. However, this interpretation explains only the slower response times in detecting central interest changes, but not the faster detection of changes in marginal interest areas observed for the emotional stimuli. A possible explanation is that the top-down attentional process toward emotion-relevant environmental aspects improves the detection performance [51,52]. However, a more convincing view, which can integrate both the above hypotheses, is that negative scenes grab attention at a very early stage [50]. Due to the aversive nature of the negative stimuli, attention could be redirected toward more positive areas of the environment. In particular, when a negative scene is presented, attention should be diverted from the attentional focus (i.e., central interest area) with a decay of the bottom-up processes that do not allow highlighting the central interest changes but, conversely, to faster detect the changes in marginal location of the scene. In this sense, negative scenes grab attention rapidly for their arousing valence and increase change blindness when bottom-up processes are involved; however, it decreases when top-down attentional processes are implicated.

Despite the general effect due to the change type being consistent across the groups, different patterns emerged considering the scene valence. Individuals with high trait anxiety showed a faster detection time in emotional scenes, especially negative, compared to neutral ones. Instead, individuals with low trait anxiety showed less bias in processing emotional scenes. These results are consistent with previous studies showing that anxiety is associated with deficits during the processing of neutral stimuli [35]. Moreover, in people with high trait anxiety than low trait anxiety, fast detection of a change in positive scenes was highlighted. This result could appear counterintuitive since research supports a causal link between attentional bias for negative and threatening stimuli and anxiety vulnerability [53,54,55]. Accordingly, people differ in their tendency to preferentially allocate attention toward negative emotional stimuli, which confers different anxiety susceptibility. However, little is known about the elaboration of positive stimuli in anxiety. Attention allocation toward positive stimuli may play a role in attenuating emotional reactivity to the environment [54,56]. According to the results of this study, individuals with high trait anxiety may exhibit a bias toward positive stimuli in line with a threat-avoidant coping strategy (i.e., attentional control theory; [5]), which would help to react to environmental stimuli that can generate emotional susceptibility.

Another result that supports an association between anxiety and attentional processes is given by the different patterns associated with the change type. Individuals with both high and low trait anxiety had a faster detection of central interest changes in neutral than emotional stimuli; however, only participants with high anxiety were able to faster detect the marginal interest changes in emotional scenes than neutral ones. These results confirm an effect of stimuli valence in anxious individuals, specifically considering the top-down attentional process. Adaptively, emotional stimuli require rapid responses, and the attentional system prioritizes them (i.e., with a perceptual benefit for negative ones) over relatively unemotional stimuli [57]. For these reasons, high and low anxious individuals showed a similar pattern in automatic attention, with higher bottom-up change blindness for emotional stimuli than neutral ones. The attentional processing of emotionally salient environmental stimuli would influence the bottom-up process in both individuals with high and low trait anxiety [58]. Another explanation can refer to the sample characteristics; subclinical conditions do not properly lead to underline differences in the automatic attentional process considering anxiety levels. In contrast to bottom-up processes, top-down processes are endogenous, voluntary, and goals-driven, playing a crucial role in developing and maintaining attentional bias in anxiety disorders [57]. According to the anticipation model [59], a cardinal feature of anxiety is the anticipation of negative future events, which determines a better scanning of the environment to search for emotionally relevant aspects. This could explain the faster detection of marginal changes, evidenced only in participants with higher trait anxiety.

Finally, our study aimed to analyze the role of HRV in the association between anxiety and change blindness. Previous studies have shown that HRV is related to cognitive functions (for a review, see [22]) such as memory [20], cognitive control [20], attention [60], and decision making [35,61]. Moreover, anxiety has been associated with low HRV [45,62]. In our study, no difference in the HRV pattern was observed between the groups with high and low trait anxiety. According to other studies (e.g., [35,48]), this result could be due to the sample’s characteristics of young participants with subclinical anxiety, but without a diagnosis of anxiety disorders. However, a moderating role of HRV in influencing the attentional processes in high anxiety conditions was expected. Our findings show that fast detection of central interest changes, but not of marginal ones, was associated with high resting-state HRV, both considering the time (RMSSD (ln)) and frequency domains (HF (ln); LF (ln)). Interestingly, these results were evidenced only in anxious individuals. Considering these results, HRV appears to have a role in the automatic attentional bottom-up process. As attentional control in everyday life requires efficient attentional focus, this result is interesting, especially considering anxiety. In line with the neurovisceral integration model [19], HRV is a predictor of cognitive control, influencing automatic and focused attentional processes. Accordingly, our results indicate that higher HF-HRV was associated with decreased response times in detecting central interest changes, regardless of trait anxiety levels. Further studies should analyze the moderation role of HRV in these relationships. This pattern could explain the absence of differences between groups in attentional performance. Subclinical anxiety levels would be characterized by an initial alteration of the autonomic activation, but not a significant impairment. This trend should compensate the attentional strategies, allowing better performances in the change detection. The autonomic alteration would be associated with an impairment of the early stages of the attentional process; when the behavior becomes goal-directed, and the voluntary top-down attentional mechanism gets involved, no drop in the performances emerges. We can conclude that vagal modulation (HRV) in the first stage of the attentional process moderates the performance, leveling any differences in performance (i.e., change blindness performance) between individuals with and without high levels of trait anxiety. HRV would affect the strength and direction of the relationship between trait anxiety and attentional processes. This finding is in line with the results of previous studies reporting that resting HRV predicts inhibitory mechanisms involved in the attentional processes. For example, some authors showed that lower HRV was associated with failure in shift attention during fearful valence tasks, whereas higher HRV demonstrated similar reactions to fearful and neutral stimuli, suggesting better attention management [60]. Moreover, higher HRV is associated with more efficient control of focused attention, critical for daily life [46]. Our findings underline an association with the detection of central interest changes, confirming these aspects.

Our study does presents some limitations. First of all, the small sample size may have compromised the statistical power of moderating results. Given this limitation, the results should be considered preliminary and replicated in larger samples. Moreover, further studies should analyze the role of HRV through more sophisticated statistical analyses (e.g., Structural equation modeling). The participants’ age range represents the second limit; the results cannot be generalized to older samples. This may be particularly relevant considering that cardiac vagal activity tends to decline with age [16], and its impairment would influence the attentional processes. Another limitation related to the participants’ characteristics is the consideration of subclinical anxiety evaluated across the sample’s percentiles. The adoption of clinical populations with anxiety diagnosis could better underline differences between groups. Moreover, in line with vagal tank theory [44], further studies should also measure HRV during the performance of tasks and recovery to underline autonomic changes better [61]. Finally, it would be interesting to deepen the relationship between HRV and the emotional flicker task to underline both HRV change related to stimuli valence and in clinical samples characterized by possible alterations in cognitive functioning also involving an attentional mechanism such as cognitive impairment [63], hypertension [64,65], and obesity [66].

## 5. Conclusions

To our knowledge, this is the first study in which the role of heart rate variability was considered in the relationship between anxiety and attention, with a paradigm that allows the analysis of both automatic and voluntary processes of attention [28] toward ecological emotional stimuli. Although our results should be taken as preliminary and need replication, they provide interesting insights. On one hand, our findings confirm the emotionality hypothesis in anxiety [67] that proposes that anxious subjects show attention deficits concerning emotional stimuli, both positive and negative. On the other hand, an association between HRV anxiety and change blindness was discovered. In individuals with high trait anxiety, greater HRV could provide a “protective effect” that should be considered within an integrated approach to reduce cognitive biases associated with anxiety disorders. These results reinforce the role of HRV as a predictor of autonomic and attentional responses.

## Figures and Tables

**Table 1:** Principal characteristics of the groups.

	Low Trait Anxiety (*n* = 36)	High Trait Anxiety (*n* = 39)	F	P
Female (%)	20 (55.6)	20 (51.3)		
Male (%)	16 (44.4)	19 (48.7)		
Age	24.5 (2.7)	23.5 (2.2)	3.15	0.08
Trait Anxiety score	28.2 (2.9)	55.5 (5.0)	821.0	0.0001 *
BMI	23.1 (3.7)	22.9 (2.2)	<1	0.90
SBP	116.5 (12.5)	121.5 (10.2)	2.16	0.14
DBP	72.9 (7.8)	71.9 (6.9)	<1	0.62
*HR*	73.9 (11.1)	76.10 (11.7)	<1	0.51
HRV indices in Resting State				
RMSSD (ms)	36.15 (13.9)	34.8 (15.6)	<1	0.63
LF (ln)	6.9 (0.6)	6.6 (0.7)	1.96	0.16
HF (ln)	6.2 (0.9)	5.9 (0.9)	1.20	0.27

BMI: Body Mass Index; SDB: Systolic Blood Pressure; DBP: Diastolic Blood Pressure; HR: Heart Rate; RMSSD: root mean square of successive differences between normal heartbeats; LF (ln): Natural logarithmic transformation of low-frequency; HF (ln): natural logarithmic transformation of high-frequency. *: *p* < 0.05

**Table 2:** Performance (RTs mean and standard deviation) in the emotional flicker task considering emotional vs. neutral stimuli.

Change Type	Stimuli Valence	Low Trait Anxiety	High Trait Anxiety
CI	Emotional	6064 (2513)	5893 (2696)
	Neutral	3821 (3128)	3992 (3135)
MI	Emotional	22,707 (8837)	19,432 (7716)
	Neutral	22,402 (8608)	25,288 (12271)
CI change		5319 (2132)	5346 (2270)
MI change		22,627 (6884)	21,356 (1103)

CI: Central interest changes; MI: Marginal interest changes.

**Table 3:** Performance (RTs mean and standard deviation) in the emotional flicker task considering the emotional valence of the stimuli and the type of change in individuals with low and high trait anxiety.

Change Type	Valence	Low Trait Anxiety	High Trait Anxiety
CI	Negative	6952 (4326)	6451 (4389)
	Neutral	3820 (3173)	3992 (3135)
	Positive	5338 (2092)	5335 (2929)
MI	Negative	16,713 (8340)	16,486 (7182)
	Neutral	22,335 (8724)	25,288 (12,271)
	Positive	28,845 (15,409)	22,378 (10,366)

CI: Central interest changes; MI: Marginal interest changes.

**Table 4:** Pearson’s r correlation between heart rate variability (HRV) indices and RTs in the flicker task considering the two groups of participants.

			High Anxiety Trait			Low Anxiety Trait		
			RMSSD (ln)	LF (ln)	HF (ln)	RMSSD (ln)	LF (ln)	HF (ln)
CI		*r*	**−0.37**	−0.30	**−0.40**	0.09	−0.20	−0.18
		*p*	**0.03**	0.083	**0.02**	0.61	0.27	0.32
MI		*r*	0.044	0.062	0.13	0.13	0.25	0.067
		*p*	0.80	0.72	0.47	0.45	0.15	0.71
CI	Negative	*r*	−0.20	−0.28	−0.23	0.01	−0.14	−0.11
		*p*	0.24	0.10	0.19	0.95	0.43	0.52
CI	Neutral	*r*	−0.32	−0.14	**−0.35**	0.06	−0.26	−0.17
		*p*	0.06	0.42	**0.04**	0.73	0.13	0.33
CI	Positive	*r*	−0.19	−0.09	−0.20	0.16	0.10	−0.04
		*p*	0.27	0.61	0.25	0.37	0.58	0.82
MI	Negative	*r*	−0.16	−0.04	−0.04	0.05	0.02	0.004
		*p*	0.36	0.82	0.81	0.77	0.90	0.98
MI	Neutral	*r*	0.021	0.06	0.10	−0.16	0.12	−0.09
		*p*	0.91	0.71	0.56	0.36	0.50	0.61
MI	Positive	*r*	0.18	0.08	0.17	0.24	0.26	0.14
		*p*	0.31	0.66	0.345	0.18	0.16	0.45

CI: Central interest changes; MI: Marginal interest changes; LTA: Low trait anxiety group; HTA: High trait anxiety group; RMSSD: root mean square of successive differences between normal heartbeats; LF (ln): Natural logarithmic transformation of low-frequency; HF (ln): Natural logarithmic transformation of high-frequency. Bold: negative correlations.

## Data Availability

Please contact the authors for data availability.

## Supplementary Materials

The following are available online at https://www.mdpi.com/article/10.3390/brainsci11040480/s1, Supplementary material: Participant Flow-chart.
