# Supplementary figures and images for: Anxiety and Attentional Processes: The Role of Resting Heart Rate Variability
Source: Brain Sci. 2021 Apr 9;11(4):480. doi: 10.3390/brainsci11040480 (PMC8070415; doi:10.3390/brainsci11040480)

Supplementary material: Participant Flow-chart

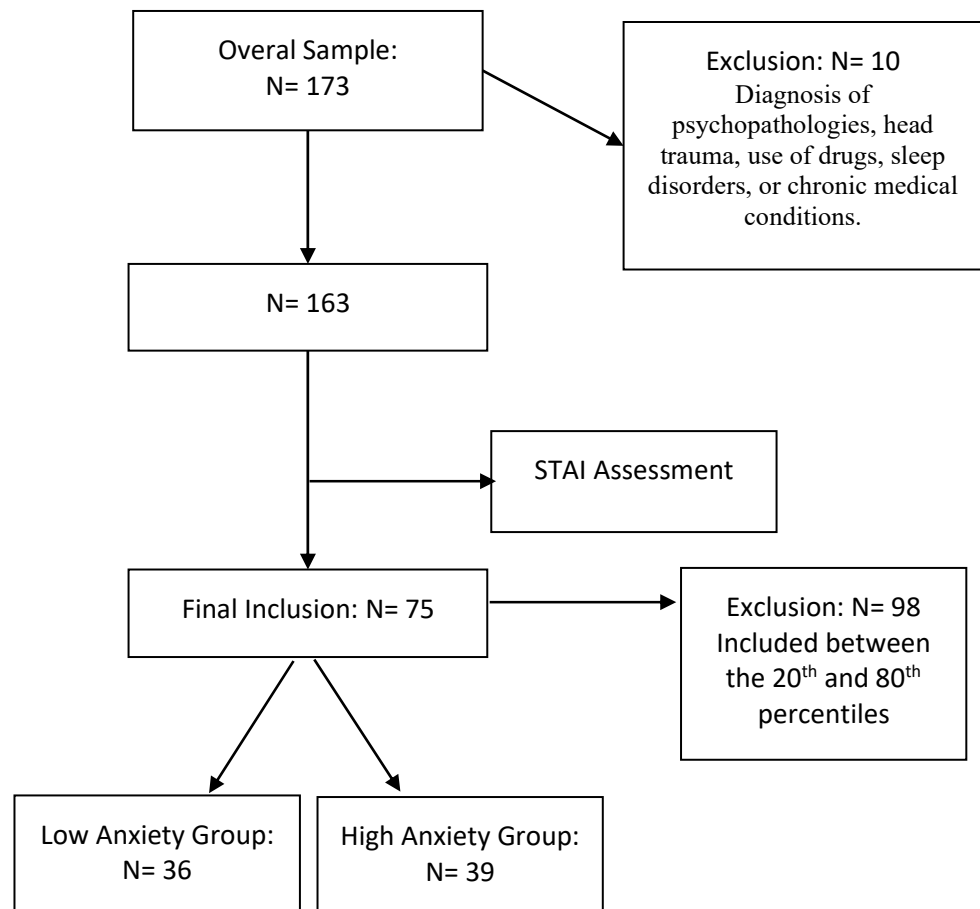

Supplement: Supplementary file 1 [file brainsci-11-00480-s001.pdf]
